# Effect of thymic stimulation of CD4+ T cell expansion on disease onset and progression in mutant SOD1 mice

**DOI:** 10.1186/s12974-015-0254-3

**Published:** 2015-02-27

**Authors:** Rebecca K Sheean, Richard H Weston, Nirma D Perera, Angela D’Amico, Stephen L Nutt, Bradley J Turner

**Affiliations:** The Florey Institute of Neuroscience and Mental Health, University of Melbourne, 30 Royal Parade, Parkville, Victoria 3052 Australia; Centre for Neuroscience, University of Melbourne, 30 Royal Parade, Parkville, Victoria 3052 Australia; The Walter and Eliza Hall Institute of Medical Research, University of Melbourne, 1G Royal Parade, Parkville, Victoria 3052 Australia

**Keywords:** ALS, SBMA, SOD1, T cells, Tregs, Androgen receptor

## Abstract

**Background:**

The peripheral immune system is implicated in modulating microglial activation, neurodegeneration and disease progression in amyotrophic lateral sclerosis (ALS). Specifically, there is reduced thymic function and regulatory T cell (Treg) number in ALS patients and mutant superoxide dismutase 1 (SOD1) mice, while passive transfer of Tregs ameliorates disease in mutant SOD1 mice. Here, we assessed the effects of augmenting endogenous CD4+ T cell number by stimulating the thymus using surgical castration on the phenotype of transgenic SOD1^G93A^ mice.

**Method:**

Male SOD1^G93A^ mice were castrated or sham operated, and weight loss, disease onset and progression were examined. Thymus atrophy and blood CD4+, CD8+ and CD4+ FoxP3+ T cell numbers were determined by fluorescence activated cell sorting (FACS). Motor neuron counts, glial cell activation and androgen receptor (AR) expression in the spinal cord were investigated using immunohistochemistry and Western blotting. Differences between castrated and sham mice were analysed using an unpaired *t* test or one-way ANOVA.

**Results:**

Castration significantly increased thymus weight and total CD4+ T cell numbers in SOD1^G93A^ mice, although Tregs levels were not affected. Despite this, disease onset and progression were similar in castrated and sham SOD1^G93A^ mice. Castration did not affect motor neuron loss or astrocytic activation in spinal cords of SOD1^G93A^ mice; however, microglial activation was reduced, specifically M1 microglia. We also show that AR is principally expressed in spinal motor neurons and progressively downregulated in spinal cords of SOD1^G93A^ mice from disease onset which is further enhanced by castration.

**Conclusions:**

These results demonstrate that increasing thymic function and CD4+ T cell number by castration confers no clinical benefit in mutant SOD1 mice, which may reflect an inability to stimulate neuroprotective Tregs. Nonetheless, castration decreases M1 microglial activation in the spinal cord without any clinical improvement and motor neuron rescue, in contrast to other approaches to suppress microglia in mutant SOD1 mice. Lastly, diminished AR expression in spinal motor neurons, which links to another motor neuron disorder, spinal bulbar muscular atrophy (SBMA), may contribute to ALS pathogenesis and suggests a common disease pathway in ALS and SBMA mediated by disruption of AR signalling in motor neurons.

## Background

Neuroinflammation consisting of resident astrocyte and microglial activation and infiltration of peripheral T cells into the central nervous system (CNS) occurs in the progression of amyotrophic lateral sclerosis (ALS), an unrelenting and fatal disease caused by selective loss of motor neurons in the brain, brainstem and spinal cord. Neuroinflammatory responses in ALS are often referred to as a double-edged sword, with both beneficial and deleterious effects on motor neuron survival [1]. Both the innate and adaptive immune systems play important roles in neuroinflammatory responses in ALS. In the CNS, the innate immune response involves activation of pro-inflammatory M1 or anti-inflammatory M2 microglia [2-4]. Microglial expression of inducible nitric oxide synthase (iNOS) or arginase 1 (Arg1) is typically used to identify M1 and M2 microglia, respectively. The adaptive immune system primarily involves the action of T cells which differ in expression of cell surface molecules and functional roles. T cells include CD8+ cytotoxic T cells which induce apoptosis of target cells and CD4+ T helper cells which suppress the effects of CD8+ T cells and M1 microglia [5]. Importantly, T cells born in the bone marrow must first migrate to the thymus where they undergo maturation to CD4+ and/or CD8+ cells [6].

T cell infiltration is widely observed in the spinal cord and cerebral cortex of ALS patients [7-11]. CD4+ T cells are recruited to sites of motor neuron degeneration such as the spinal cord ventral horns and corticospinal tracts [12]. Studies in transgenic mice expressing mutant superoxide dismutase 1 (SOD1) linked to ALS have yielded important insights into the role of T cells in ALS progression. SOD1^G93A^ mice on recombination-activating gene 2 (*RAG2*) or *CD4* knockout backgrounds, both ablating functional CD4+ T cells, showed accelerated disease progression and altered microglial activation [13]. Furthermore, deletion of T cell receptor β chain to prevent T cell maturation also accelerated disease progression in SOD1^G93A^ mice [14]. These studies suggest that specific T cell subtypes may confer protection in ALS models. Recently, the T cell subtype responsible for neuroprotection in ALS, CD4+ CD25+ FoxP3+ regulatory T cells (Tregs), was identified by passive transfer of Tregs into SOD1^G93A^ mice prolonging lifespan [15,16]. Tregs suppress M1 microglia activation and CD8+ T cell and CD4+ CD25− T effector cell expansion which would promote motor neuron survival [17-19].

The T cell population is maintained by differentiation and release of mature T cells at the thymus into circulation. The thymus undergoes atrophy and inactivation with normal ageing which is driven by circulating androgens released from puberty [6]. It was recently shown that thymus inactivation is significantly accelerated with reduced CD4+ T cell numbers in the blood of ALS patients and mutant SOD1 mice [20,21]. In particular, Treg numbers are reduced in ALS patients which correlate with increased disease severity [21]. These results suggest that a chronic deficiency of thymic function and CD4+ T cells occurs in ALS which may potentiate neuroinflammation, motor neuron loss and disease progression. Therefore, strategies that augment thymic function and CD4+ T cell production may ameliorate ALS progression. Here, we tested the effects of thymic stimulation on the disease phenotype of SOD1^G93A^ mice using castration which is well known to both activate the thymus and boost T cell levels.

## Methods

### Ethics statement

All experiments conformed to the Australian National Health and Medical Research Council published code of practice and were approved by the Howard Florey Institute Animal Ethics Committee (permit number 12–012).

### Animals and castration surgery

Transgenic SOD1^G93A^ mice derived from the B6SJL-TgN(SOD1*G93A)1Gur/J line (Jackson Laboratory, Bar Harbor, ME) were backcrossed and maintained on a pure C57BL/6 background. Prepubescent (5-week-old) male mice were anesthetised via isoflurane using a gas anaesthetic machine and either castrated (Cx) or sham operated. A shallow 1-cm incision was made along the ventral midline of the scrotum. The tunica was exposed, and a small 5-mm incision was made at the base and the testis exposed. The spermatic artery and vas deferens were sutured to prevent bleeding before removing the testis by cutting below the ligature. After removing both testes, the scrotum was closed with sutures and Michel clips (Fine Science Tools, North Vancouver, British Columbia, Canada). Mice were injected with meloxicam (3 mg/kg, SC) for analgesia and warmed to recovery under a heat lamp. Surgical instruments were sterilised by autoclave prior to surgery. Age-matched and intact C57BL/6 wild-type (WT) mice were used as controls for immunoblotting and immunohistochemistry. For androgen receptor (AR) immunoblotting, separate intact WT and SOD1^G93A^ males aged postnatal day 60 (P60), P90 and P120 were used. Mice were killed by lethal injection (sodium pentobarbitone, 100 mg/kg, IP).

### Behavioural analysis

Mouse weight and locomotor function were analysed weekly from P60 using an accelerating Mouse Rota-Rod 47600 (Ugo Basile, Monvalle, Italy). Mice were trained at P60 by one constant speed (16 rpm) over 5 min and two ramping sessions at 3 to 30 rpm over 5 min with 10-min rest intervals between each training session. The next day, mice were tested twice at 3 to 30 rpm over 5 min and the average latency to fall in seconds was recorded. Disease onset was retrospectively determined using the age of peak body weight preceding denervation muscle atrophy onset as previously described [22]. Onset of motor deficit was determined using the age of peak rotarod activity preceding first impairment. For survival analysis, mice were killed by lethal injection at clinical end point defined by onset of hindlimb paralysis.

### Histology

Mice were transcardially perfused with PBS followed by 4% paraformaldehyde (PFA) in 0.1 M phosphate buffer. Lumbar spinal cords were dissected out, postfixed in 4% PFA for 2 h and cryoprotected in 30% sucrose in PBS overnight at 4°C. Cords were embedded in optimal cutting temperature medium by freezing in isopentane cooled by liquid nitrogen. Horizontal 20-μm sections were serially cut and stained with 0.5% cresyl violet using a standard protocol, dehydrated and coverslipped. Nissl-positive motor neurons in every third section were counted from a total of 60 ventral horns per mouse (*n* = 3 to 4 mice per group) identified by neuronal morphology and size exceeding 20-μm diameter with a distinct nucleolar profile.

### Immunohistochemistry

Spinal cord sections were permeabilised in 0.4% TX-100 in PBS for 10 min; blocked in 5% skim milk in PBS for 30 min and incubated with mouse NeuN (1:1,000, Millipore, MAB377, Bayswater, VIC, Australia), mouse glial fibrillary acidic protein (GFAP) (1:200, Millipore, MAB360), mouse CD11b (1:100, MCA275GA, Serotec, Raleigh, NC, USA) or rabbit AR (1:100, Millipore, 06–080) antibodies overnight at 4°C. For M1 and M2 microglial staining, sections were incubated with mouse iNOS (1:100, sc-7271, Santa Cruz Biotechnology, TX, USA) or mouse Arg1 (1:100, sc-271430, Santa Cruz Biotechnology,) and goat Iba1 (1:200, ab107159, Abcam, Cambridge, UK). Sections were incubated with Alexa Fluor-conjugated secondary antibodies (1:200, Molecular Probes, Life Technologies, Mulgrave, VIC, Australia) for 2 h, stained with Hoechst 33342 (1:10,000, Invitrogen) for 15 min and mounted using fluorescent mounting medium (Dako, North Sydney, NSW, Australia) for microscopy using an Olympus FV 1000 (Olympus, Notting Hill, VIC, Australia) confocal microscope. Representative images are shown from *n* = 3 to 4 mice per group. Images were captured using identical exposure and gain settings. Negative controls without primary antibodies were performed which produced no staining.

### Immunoblotting

Fresh-frozen lumbar spinal cords were homogenised in RIPA lysis buffer containing 50 mM Tris-Cl, pH 7.4, 150 mM NaCl, 0.1% SDS, 1% sodium deoxycholate, 1% TX-100 and 1% protease inhibitor cocktail (Sigma-Aldrich, Castle Hill, NSW, Australia) by sonication at 50% output for 15 s; stored on ice for 20 min and centrifuged at 15,800 *g* for 20 min to collect supernatants. Proteins (20 μg) were electrophoresed through 12.5% SDS-polyacrylamide gels and transferred to Immobilon PVDF-FL membranes (Millipore). Membranes were blocked with 5% skim milk in TBST, pH 8.0, for 30 min and incubated with sheep SOD1 (1:4,000, 574597, Millipore), rabbit AR (1:500) or mouse β-actin (1:2,000, Sigma, A5316) antibodies overnight at 4°C. Blots were incubated with IRDye-conjugated secondary antibodies (1:10,000, Li-Cor, Lincoln, NE, USA) and imaged using the Odyssey Classic near infrared detection system. Blots were quantified by taking the mean grey value of SOD1 and AR bands normalised to β-actin level after subtracting background intensity.

### Fluorescence activated cell sorting analysis

Peripheral blood (approximately 200 μl) from mice (*n* = 4 per group) was collected at 2 weeks post-surgery and analysed for B cell, T cell and total cell number using density centrifugation and fluorescence activated cell sorting (FACS). Blood samples stored on ice were pulsed and resuspended in 1.5 ml mouse tonicity (MT)-PBS. Samples were underlaid by 1.5 ml Histopaque 1077 (Sigma) and centrifuged at 2,000 rpm for 20 min to allow separation of cells. The mononuclear layer was recovered and washed in MT-PBS and diluted four- to fivefold. A 10-μl sample was added to an equal volume of eosin and cells counted using a haemocytometer. Cells were transferred to glass conical tubes and centrifuged at 1,700 rpm for 7 min and incubated with 20 μl of an antibody cocktail of rat CD4-FITC (Molecular Probes), CD8-APC (ProZyme, Hayward, CA, USA), B220-Alexa 680 (ProZyme), GR-1-PeCy7 and M1-70-Pe on ice for 25 min. Following incubation, 1 ml KDS-BSS (balanced salt solution, 4-(2-hydroxyethyl)-1-piperazineethanesulfonic acid (HEPES)-buffered MT buffer, pH 7.22, containing 1.68 M NaCl, 1.68 M KCl, 1.12 M CaCl_2_, 1.68 M MgSO_4_, potassium phosphate buffer, buffered with 1.68 M HEPES made up in Milli-Q H_2_O, osmolarity 308 to 310) with 3% FCS was added with 0.2 ml FCS underlay and cells washed. Cells were resuspended in 100 μl KDS-BSS with 3% FCS + propidium iodide (0.1 μg/ml, Calbiochem) and analysed by LSR II flow cytometer (BD Biosciences, North Ryde, NSW, Australia) using 50,000 events. Splenocytes were used for single colour compensation controls. Total T cell number was determined using CD4 and CD8 marker expression which is restricted to T cells in mice, except for conventional DCs which do not circulate in peripheral blood and are selected against by lymphocyte gating.

### Statistical analysis

Data are expressed as mean ± standard error of the mean (SEM). Blood cell counts, thymus weight, Western blot densitometry, disease onset and rotarod deficit were analysed by unpaired *t* test. Neuronal counts were analysed using one-way ANOVA. Survival data were analysed using Kaplan-Meier survival analysis with the log-rank test. Analyses were performed using GraphPad Prism 5.0 software (GraphPad, La Jolla, CA, USA).

## Results

### Castration blocks thymus atrophy and increases CD4+ T cell number in mutant SOD1 mice

**Figure 1:**
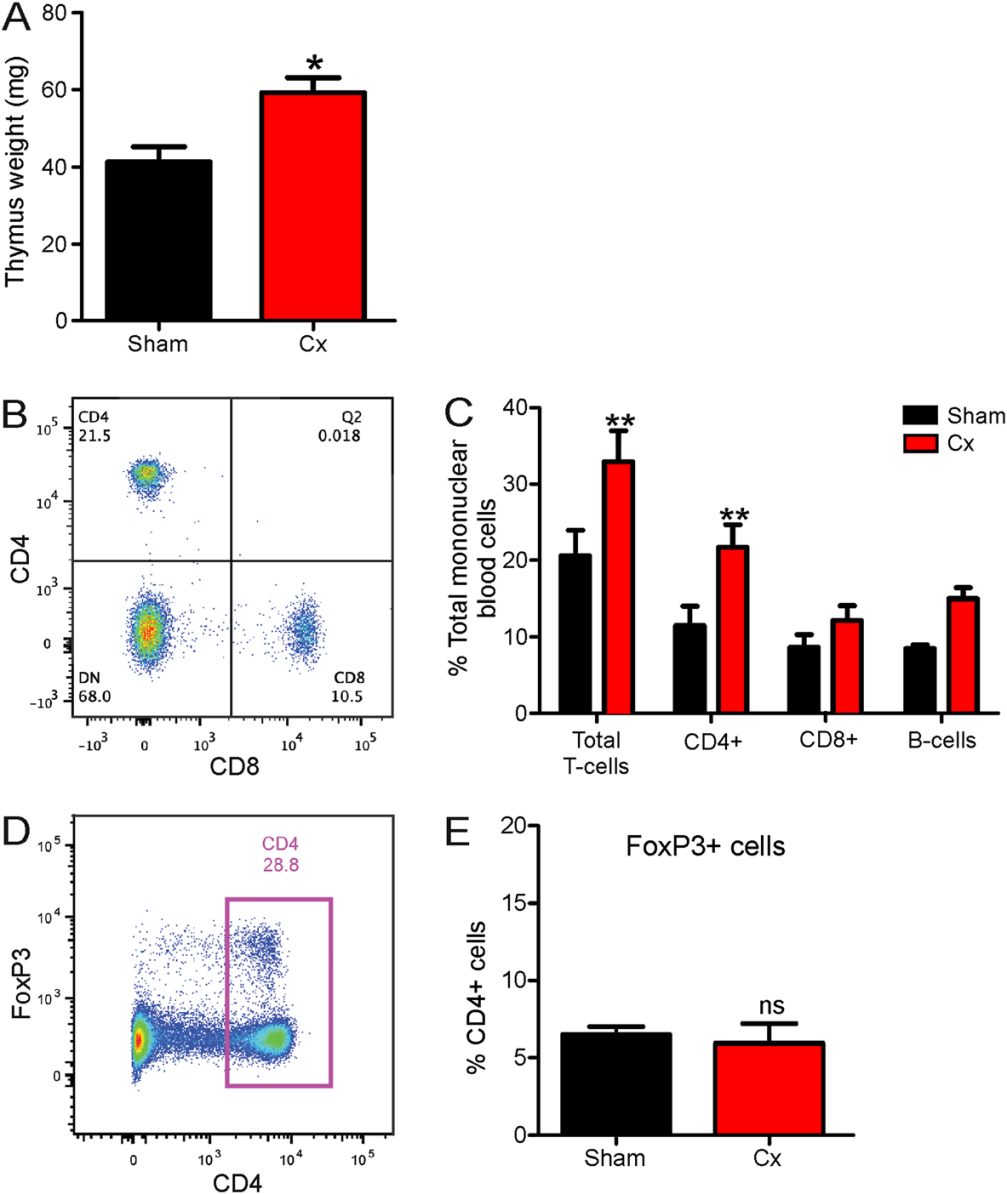
**Castration stimulates thymus growth and CD4+ T cell production in male SOD1** ^**G93A**^ **mice. (A)** Wet weight of thymus of sham and castrated (Cx) SOD1^G93A^ mice at 120 days. Data are mean ± SEM, *n* = 5, *P < 0.05 compared to sham group. **(B)** Representative scatter plot of flow cytometric analysis of CD4+ and CD8+ T cells from the blood of mice demonstrating the gates used and percentage of positive cells. **(C)** FACS analysis of blood cells at 2 weeks post-castration showing total T cell, CD4+ T cell, CD8+ T cell and B cell counts (monocytes and macrophages not shown) for sham and Cx mice, *n* = 4. **P < 0.01 compared to sham group. **(D)** Representative scatter plot of flow cytometric analysis of CD4+ and FoxP3+ T cells from blood of mice demonstrating the gates used and percentage of positive cells. **(E)** FACS analysis of blood CD4+ FoxP3+ T cell (Treg) counts for sham and Cx mice, *n* = 4.

We tested the effects of thymic stimulation on the disease phenotype of male transgenic SOD1^G93A^ mice using surgical castration. Castrations were performed on 5-week-old mice which is the time of peak thymus activity preceding puberty. Castration resulted in a 50% increase in thymus weight of mice compared to the sham group (*P* < 0.05), confirming androgen deprivation (Figure 1A). We next determined circulating T cell numbers in mice, and representative FACS plots for CD4+ and CD8+ T cells are shown (Figure 1B). Consistent with thymic stimulation, the total T cell number was significantly increased in the blood of castrated mice (*P* < 0.01, Figure 1C). This resulted from a significant twofold increase in CD4+ T cell number (*P* < 0.01), while CD8+ T cell and B cell numbers were not significantly affected by castration.

### Thymic stimulation does not alter disease progression or survival in mutant SOD1 mice

**Figure 2:**
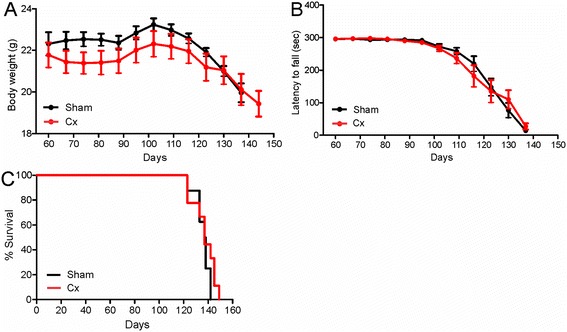
**Effect of thymic stimulation by castration on disease onset, progression and survival in male SOD1** ^**G93A**^ **mice. (A)** Castrated (Cx) mice showed a consistent reduction in body weight throughout life compared to the sham group, although not statistically significant. **(B)** Motor performance of Cx and sham mice determined by weekly rotarod test was similar. **(C)** Survival of Cx and sham mice was similar. Data are mean ± SEM, *n* = 10.

We next examined whether thymic stimulation of CD4+ T cell production alters clinical disease onset, progression and lifespan in male SOD1^G93A^ mice. As shown in Figure 2A, castrated SOD1^G93A^ mice weighed consistently less than shams, as expected following androgen removal. However, disease onset determined by the age of peak body weight preceding muscle wasting was similar in both groups (Cx 100 ± 5 days, sham 97 ± 5 days, mean ± SEM. The onset of locomotor dysfunction determined by the age of peak rotarod performance was also similar between groups (Cx 68 ± 8 days, sham 77 ± 4 days, Figure 2B). Lastly, survival, defined by age of onset of hindlimb paralysis, was not significantly affected by castration in mice (Cx 136 ± 2 days, sham 137 ± 3 days, Figure 2C). Hence, stimulation of thymic activity and CD4+ T cell number does not change disease onset or progression in male SOD1^G93A^ mice.

To explore why increased CD4+ T cell number induced by castration did not improve the disease phenotype of mice as we hypothesised, we counted Treg numbers. A representative FACS plot for CD4+ FoxP3+ T cells is shown (Figure 1D). Despite boosting CD4+ T cell number overall, FoxP3+ Treg levels were not significantly changed by castration (Figure 1E).

### Thymic stimulation does not affect spinal motor neuron loss, but reduces microglial activation in mutant SOD1 mice

**Figure 3:**
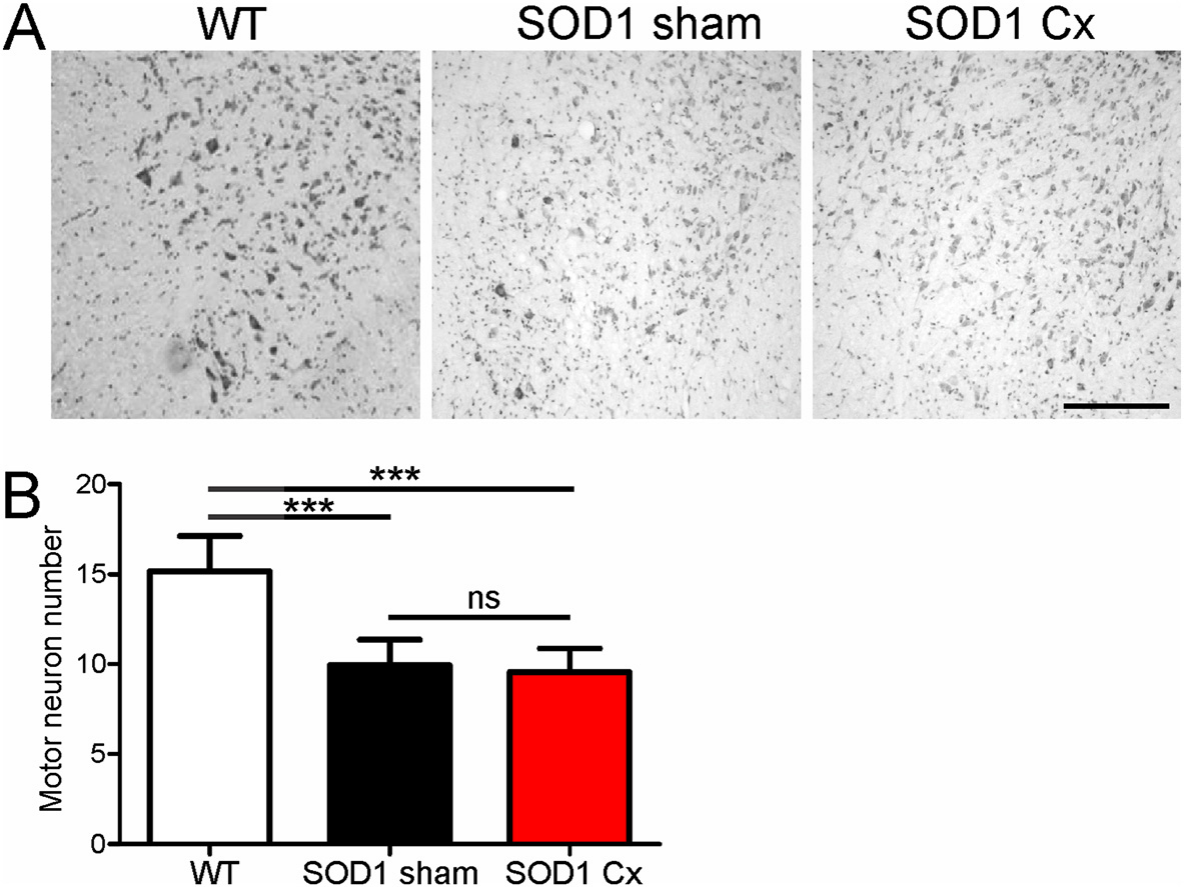
**Effect of thymic stimulation by castration on motor neuron loss in spinal cords of male SOD1** ^**G93A**^ **mice. (A)** Representative photomicrographs of ventral horns of lumbar spinal cords of wild-type (WT) or sham and castrated (Cx) SOD1^G93A^ mice stained with cresyl violet at 120 days. Scale bar = 100 μm. **(B)** Quantification of average spinal motor neuron number per ventral horn from (A) is shown. Data are mean ± SEM, *n* = 3 to 4.

To determine whether thymic stimulation affects spinal cord pathology, we quantified spinal motor neuron numbers in castrated and sham mice at P120 (Figure 3A, B). In sham SOD1^G93A^ mice, there was an approximately 50% loss of motor neurons compared to age-matched WT mice. Castration did not affect the severity of motor neuron loss in SOD1^G93A^ mice.

**Figure 4:**
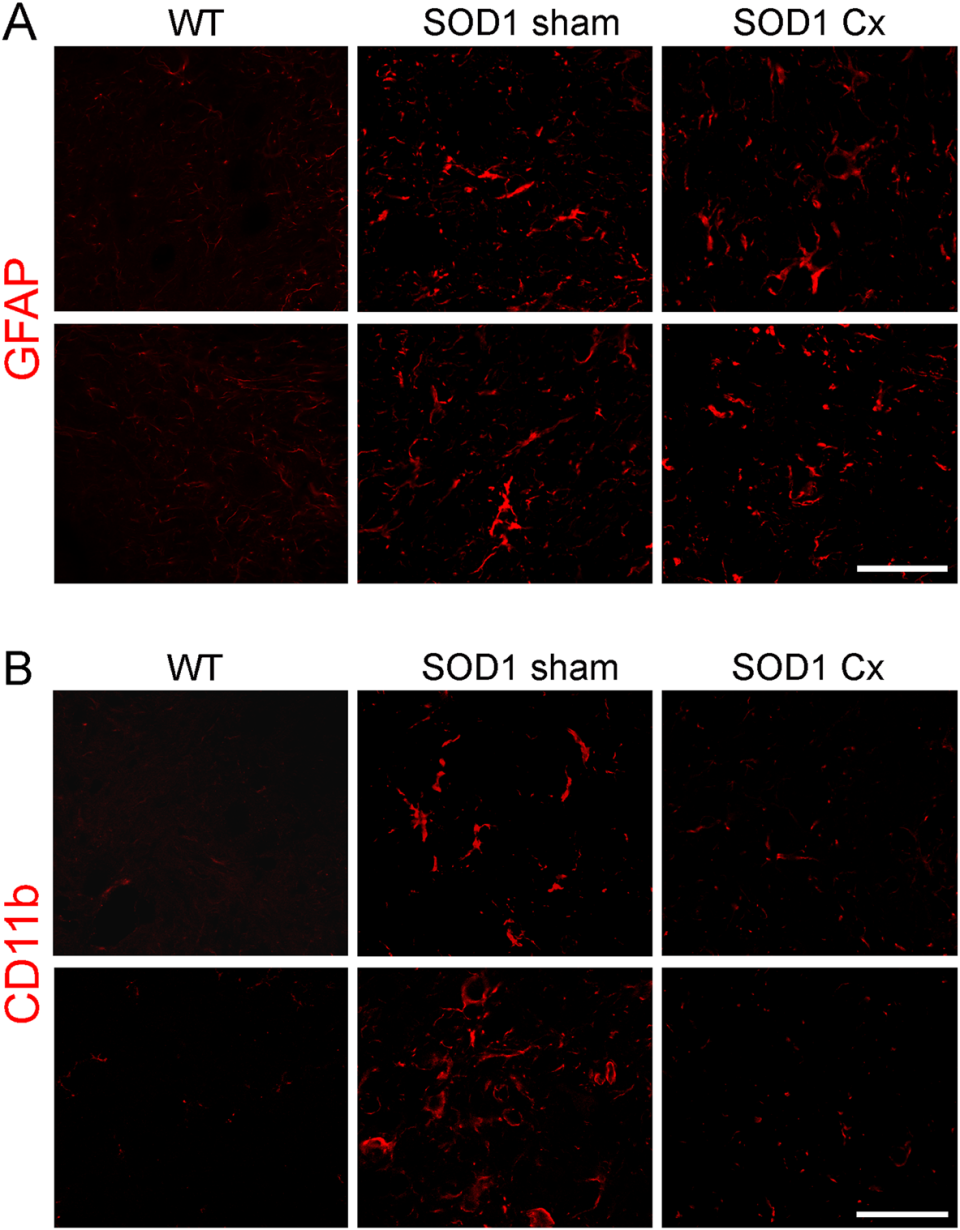
**Effect of thymic stimulation by castration on glial cell activation in spinal cords of male SOD1** ^**G93A**^ **mice.** Immunohistochemical analysis of lumbar spinal cords of wild-type (WT) or sham and castrated (Cx) SOD1^G93A^ mice at 120 days for **(A)** GFAP and **(B)** CD11b. Astrocytic activation identified by GFAP is increased in sham SOD1^G93A^ mice, but not affected by castration. Microglial activation shown by CD11b is increased in sham SOD1^G93A^ mice and reduced by castration. Scale bar = 30 μm. Representative photomicrographs from two mice per group are shown.

Astrocyte activation was next assessed using the marker GFAP. In sham SOD1^G93A^ mice, there was a pronounced astrocyte activation relative to WT mice in the spinal cord (Figure 4A). Astrocyte activation was similar in castrated and sham SOD1^G93A^ mice. Microglial activation measured using the marker CD11b was increased in sham SOD1^G93A^ mice compared to WT animals (Figure 4B). However, microglial activation was markedly reduced by castration in SOD1^G93A^ mice, consistent with the mitigating effects of increased CD4+ T cells overall on the microglia phenotype.

**Figure 5:**
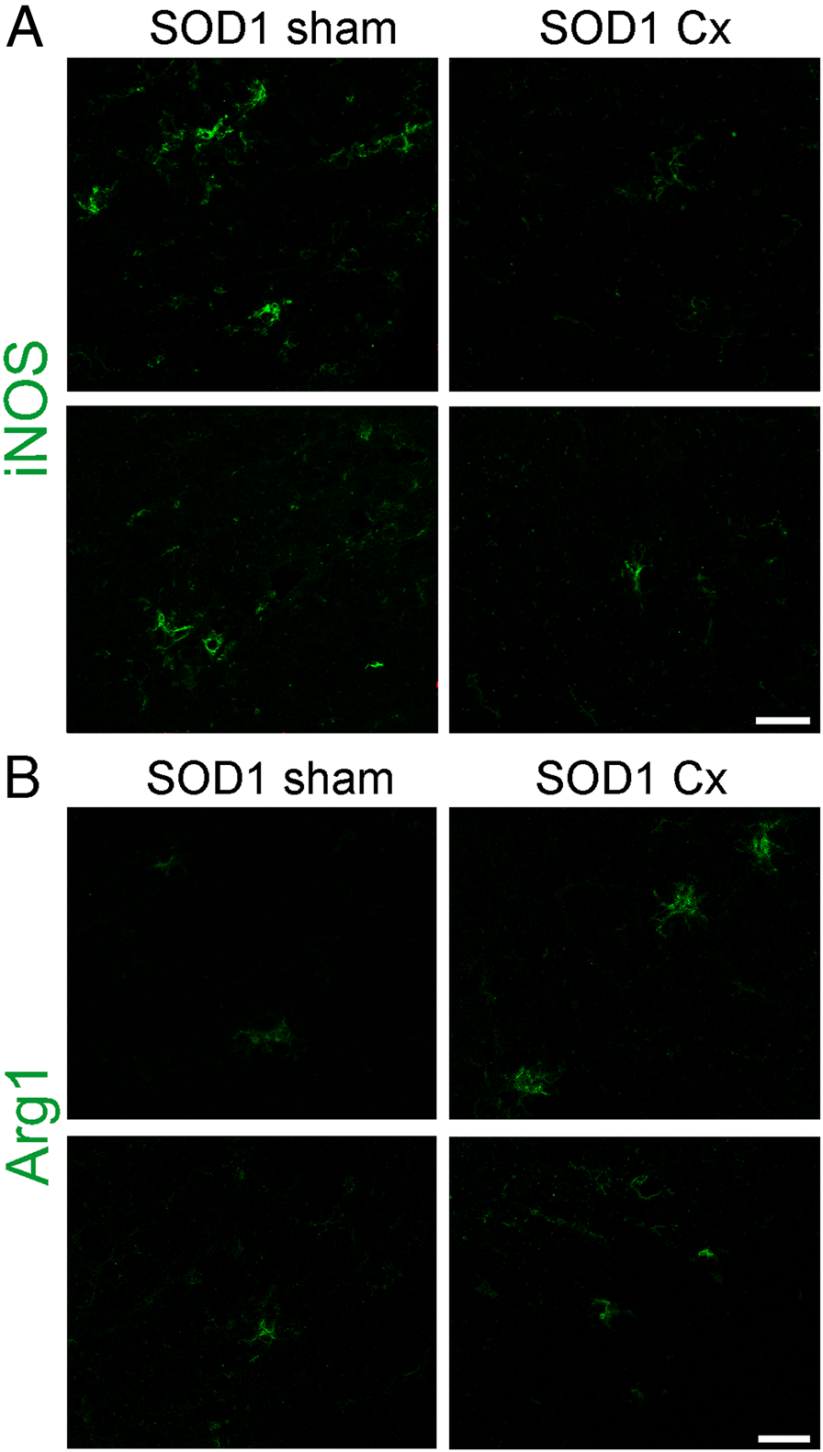
**Effect of thymic stimulation by castration on M1 and M2 microglial activation in spinal cords of male SOD1** ^**G93A**^ **mice.** Immunohistochemical analysis of lumbar spinal cords of sham and castrated (Cx) SOD1^G93A^ mice at 120 days for **(A)** iNOS and **(B)** Arg1. M1 microglial activation shown by iNOS in sham SOD1^G93A^ mice is reduced by castration. M2 microglial activation shown by Arg1 in sham SOD1^G93A^ mice is increased by castration. Microglia were identified by Iba1 (not shown). Scale bar = 20 μm. Representative photomicrographs from two mice per group are shown.

To further characterise the phenotype of microglia, we used iNOS and Arg1 as markers of M1 and M2 microglia as previously described in mutant SOD1 mice [23]. iNOS-positive microglia were identified in spinal cords of sham SOD1^G93A^ mice and were clearly reduced by castration (Figure 5A), suggesting attenuated M1 microglial activation. Conversely, Arg1-positive microglia appeared more prominent in spinal cords of castrated SOD1^G93A^ mice, compared to the sham group (Figure 5B). This implies that castration may affect the phenotype of microglia, promoting less pro-inflammatory microglia in spinal cords of mutant SOD1 mice.

### Castration and mutant SOD1 reduce androgen receptor expression in spinal motor neurons

**Figure 6:**
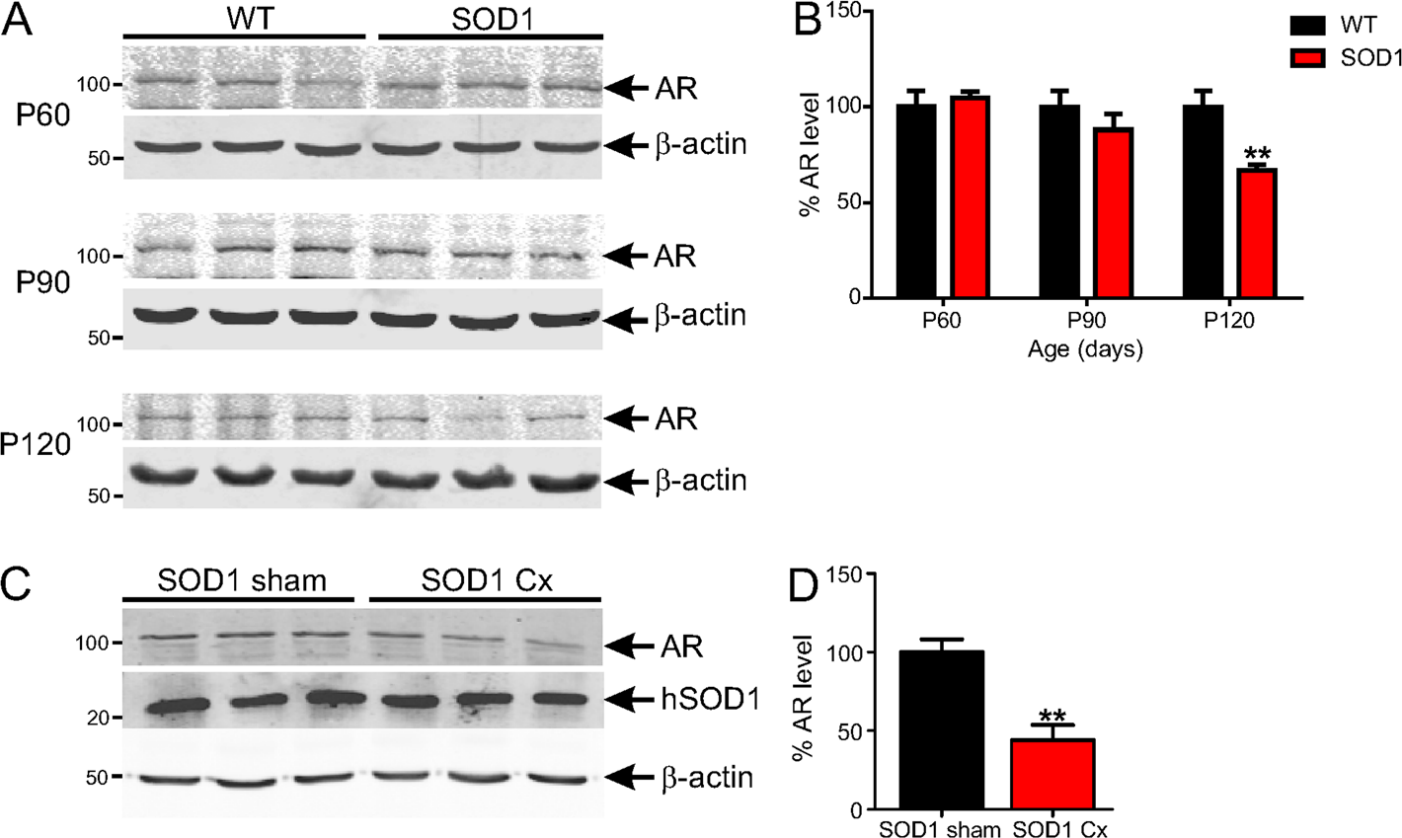
**Castration and mutant SOD1 expression reduces androgen receptor protein level in spinal cords of SOD1** ^**G93A**^ **mice. (A)** Western blot analysis of AR expression levels in lumbar spinal cords of wild-type (WT) and SOD1^G93A^ mice at 60, 90 and 120 days. **(B)** Quantification of AR expression from (A) is shown. Data are mean ± SEM normalised to β-actin and expressed as percentage of WT mice. *n* = 4, *P < 0.05 compared to WT mice. **(C)** Western blot analysis of AR and SOD1 expression in lumbar spinal cords of sham and castrated (Cx) SOD1^G93A^ mice at 120 days. **(D)** Quantification of AR expression from (C) is shown. Data are mean ± SEM normalised to β-actin and expressed as percentage of sham mice. *n* = 4 to 5, **P < 0.01 compared to sham group.

We next assessed AR expression level and cellular localisation in spinal cords of SOD1^G93A^ mice in response to castration. AR expression level was first measured in spinal cords of intact WT and SOD1^G93A^ mice at presymptomatic P60, disease onset P90 and symptomatic P120 time points. An approximately 100-kDa band corresponding to AR was detected in spinal cords of mice by Western blot analysis (Figure 5A). AR protein levels were similar in mice at P60 (Figure 6A, B). AR expression was reduced by 12% at P90 and 33% at P120 (*P* < 0.01), suggesting that mutant SOD1 leads to progressive downregulation of AR from disease onset. Castration resulted in a significant further 50% depletion of AR protein in SOD1^G93A^ mice compared to shams (*P* < 0.01, Figure 6C, D), reflecting androgen deprivation. Castration did not affect mutant SOD1 transgene expression level in spinal cords of mice (Figure 6C).

**Figure 7:**
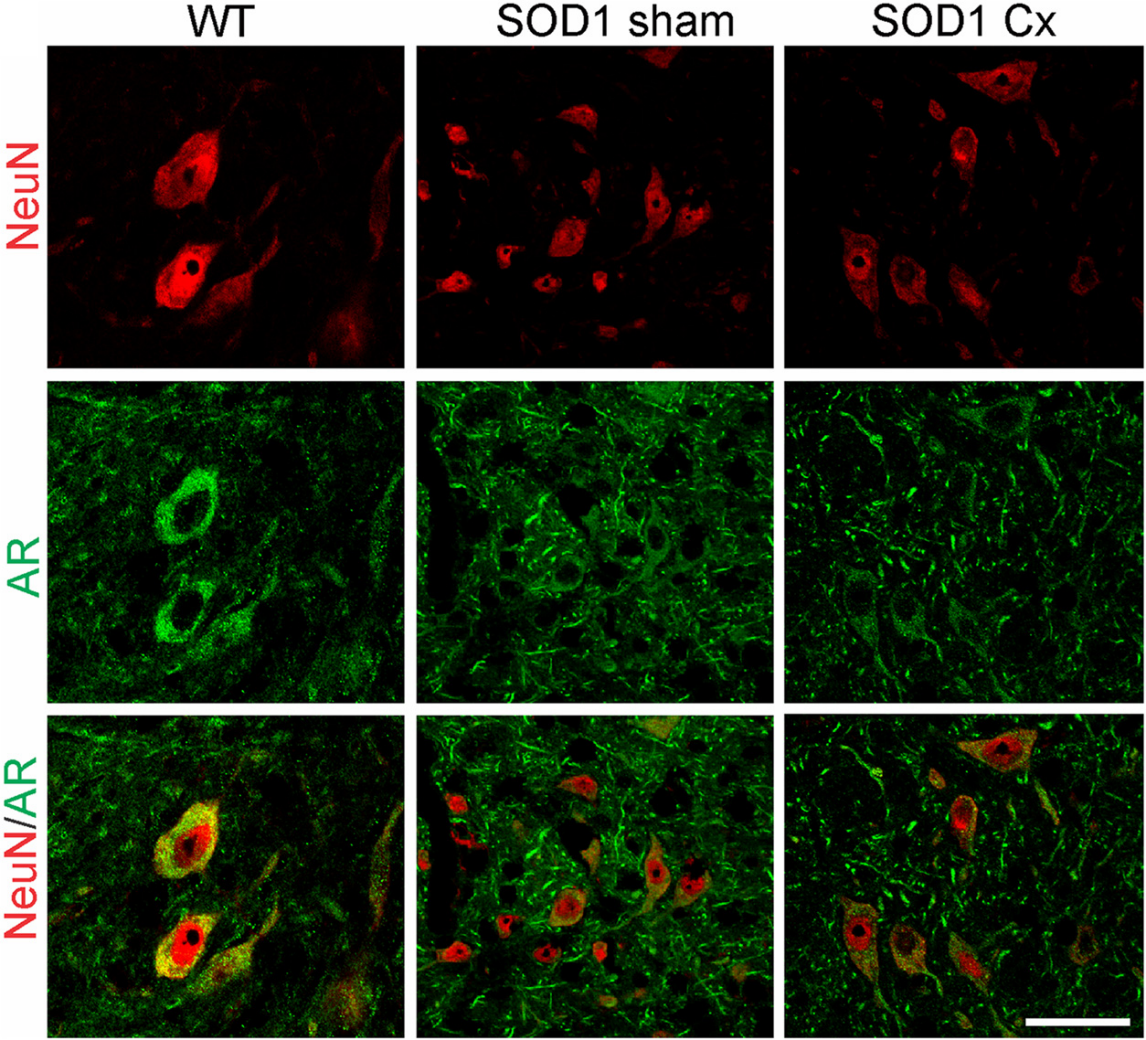
**Androgen receptor expression is reduced in spinal motor neurons by mutant SOD1 action and castration in mice.** Immunohistochemical analysis of lumbar spinal cords of wild-type (WT) or sham and castrated (Cx) SOD1^G93A^ mice at 120 days for androgen receptor (AR) and motor neurons (NeuN). AR is mainly localised within the cytoplasm of motor neuron cell bodies in WT mice. AR expression is reduced in motor neurons of SOD1^G93A^ mice and redistributed to axons and/or dendrites and further depleted in Cx SOD1^G93A^ mice. Scale bar = 30 μm.

The cellular distribution of AR was next studied in spinal cord sections. In WT mice, AR was highly expressed in motor neuron cell bodies identified by size, ventral horn location and NeuN immunoreactivity (Figure 7). AR expression was reduced in motor neuron cell bodies of sham SOD1^G93A^ mice and appeared redistributed to axons and/or dendrites. Castration of SOD1^G93A^ mice further reduced AR expression on somas of motor neurons, in line with our Western blot results (Figure 6).

**Figure 8:**
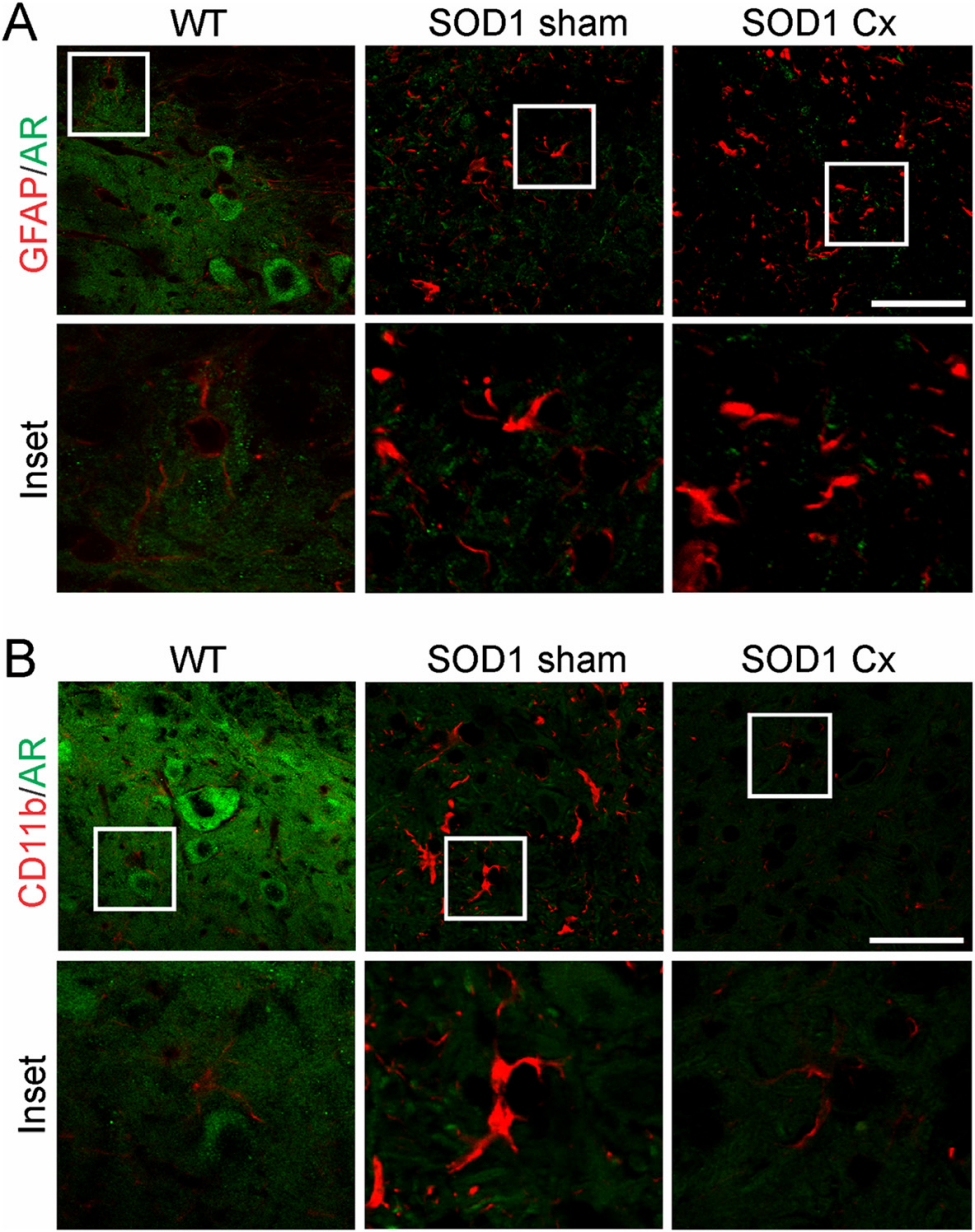
**Androgen receptor is mainly expressed by motor neurons in spinal cords of normal or mutant SOD1 mice.** Immunohistochemical analysis of lumbar spinal cords of wild-type (WT) or sham and castrated (Cx) SOD1^G93A^ mice at 120 days for androgen receptor (AR) and **(A)** GFAP or **(B)** CD11b. AR is mainly localised to motor neurons evident by cell size and location, but not astrocytes or microglia in WT or sham and Cx SOD1^G93A^ mice. Scale bar = 30 μm.

To address whether AR is expressed by glial cells in healthy or SOD1^G93A^ mice, we studied its localisation using GFAP or CD11b double immunofluorescence in the spinal cord. In WT mice, AR was not detected in astrocytes, but clearly expressed in neighbouring motor neurons (Figure 8A). In sham SOD1^G93A^ mice, activated astrocytes did not express AR and this was not affected by castration. Furthermore, in WT mice, AR was not detected in microglia, but again was abundant in adjacent motor neurons (Figure 8B). Activated microglia did not express AR in sham or castrated SOD1^G93A^ mice. Thus, AR is predominantly expressed by motor neurons in the spinal cord, consistent with selective neuronal vulnerability in SBMA, while both mutant SOD1 action and castration lead to its depletion, reminiscent of SBMA.

## Discussion

Previous studies have implicated adaptive immune system dysfunction in ALS patients and mutant SOD1 mice, showing accelerated thymus inactivation or involution, reduced CD4+ T cell number, spleen shrinkage and lymphopenia [15,16,20,21]. Whether thymus dysfunction and related deficiency of mature T cells are responsible or reactive in neurodegeneration in ALS remains unclear from these studies. To determine whether increased thymic activity and T cell production can modulate the disease phenotype of mutant SOD1 mice, we stimulated thymic function in presymptomatic SOD1^G93A^ mice using castration. Chemical or surgical castration is well proven to promote thymic function by depriving circulating androgens that mediate age-dependent atrophy of the thymus from puberty [6]. Furthermore, castration can remarkably rejuvenate the thymus in pubescent or aged mice, leading to sustained production of mature T cells throughout life [24]. We chose to castrate male SOD1^G93A^ mice at 5 weeks which precedes both puberty and clinical symptoms to maximise the effects of thymic stimulation of mature T cell production on disease. Castration significantly opposed thymus atrophy induced by androgens and disease progression in our study. This is consistent with another report showing increased thymus growth as early as 2 weeks post-castration in WT mice [25]. We also demonstrated a doubling of CD4+ T cell number at 2 weeks post-castration, concordant with other reports in mice [6,25]. Interestingly, we potentiated CD4+ T cell output, but not of CD8+ T cells, by castration in SOD1^G93A^ mice. CD4+ T cells are recruited early and linked to protection of neurons in models of axotomy [26], Parkinson’s disease [27] and ALS [28], while CD8+ T cells are present in affected tissue in end-stage disease in ALS and respond mainly to injury [28].

Despite boosting total CD4+ T cell number, thymic stimulation of SOD1^G93A^ mice did not delay disease onset or extend lifespan in our study. This contrasts with other reports using passive transfer of CD4+ T cells or Tregs to genetically T cell-deficient and bone marrow-ablated SOD1^G93A^ mice which significantly prolonged lifespan [15,16]. Various discrepancies may account for differences in results observed between our studies, namely the high purity, dose (up to 2 × 10^8^ cells repeatedly injected) and *ex vivo* activation of T cells in some of these studies, in contrast to our induction of natural thymic T cell production by castration. Another difference to these passive transfer experiments was the ablation of endogenous T cells expressing mutant SOD1 and reconstitution with wild-type or SOD1^G93A^ T cells [15,16], whereas our study induced mutant SOD1 expressing T cells on an intact adaptive immune system background. Another possibility is that castration-induced effects on the thymus may be transient, leading to increased thymus size and CD4+ T cell production that could eventually decline with age [29], although the increased weight of the thymus at end stage in SOD1^G93A^ mice argues against this.

One key difference from our study is that castration was unable to promote expansion of Tregs, which are linked to neuroprotection in ALS. Tregs which comprise 5% to 10% of peripheral CD4+ T cells [30] may be essential for slowing disease progression in mutant SOD1 mice. However, our findings are consistent with other studies showing that physical or chemical castration results in increased thymic output that is predominantly in the form of naive T cells [6,24,25]. Another possible explanation for the inability of castration to expand Treg number may result from the different homeostatic mechanisms between naive CD4+ T cells and Tregs. Tregs constitutively express the interleukin 2 receptor (IL-2R) and are more responsive and dependent on IL-2 than naive T cells which lack the alpha chain of IL-2R (CD25) [31]. While castration increases thymic output, homeostatic control of T cell numbers is not uniform and Treg numbers are limited by IL-2 availability, unlike naive cells. Disease progression in mutant SOD1 mice likely further decouples Tregs from total T cell numbers, as there is evidence that Treg numbers are reduced in ALS patients [21].

Interestingly, we observed a marked reduction in microglial activation, specifically M1 microglia, in spinal cords of castrated SOD1^G93A^ mice which would imply an attenuation of neuroinflammation independently of Tregs. Thus, other CD4+ T cell subtypes may also modulate microglial activation in ALS models. Surprisingly, the marked reduction in microgliosis did not confer any benefit to clinical progression and motor neuron survival in mutant SOD1 mice. This contrasts with previous studies showing delayed disease progression in mutant SOD1 mice with suppression of M1 microglial activation or phenotype [16]. While this discrepancy most likely reflects the different approaches used to modulate microglial activation and degree of modulation, our results suggest that inhibition of microglial activation as a treatment goal may not be as straightforward in this mouse model of ALS.

Our evidence showing no effect of castration on disease onset and progression in SOD1^G93A^ mice is consistent with a recent report using SOD1^G93A^ rats [32]. Gonadectomy of presymptomatic male or female SOD1^G93A^ rats did not affect disease onset or survival, nor did treatment with the steroid dehydroepiandrosterone [32]. In contrast, treatment of castrated male SOD1^G93A^ mice with an anabolic steroid, nandrolone decanoate, worsened clinical outcome, although castration alone did not significantly modify survival [33]. Furthermore, administration of dihydrotestosterone to male SOD1^G93A^ mice promoted muscle growth and strength and led to modest improvements in motor function and lifespan [34]. Despite this, ovariectomy of SOD1^G93A^ mice hastened disease progression which was rescued by 17β-estradiol therapy [35]. Hence, androgen deprivation induced by castration in male SOD1^G93A^ rats [32] and mice here does not affect disease course, suggesting that factors other than circulating androgens may account for the increased incidence of ALS in males than females in humans [36] and reduced survival of male SOD1^G93A^ mice compared to females [37]. However, androgen and oestrogen therapy in SOD1^G93A^ mice is protective, possibly by stimulating muscle growth and strength, independent of motor neuron survival. Interestingly, castration of male TDP-43^A315T^ mice did not significantly impact on survival [38], arguing against an androgen-dependent disease phenotype in these mice.

One potentially important finding from our study was that AR expression in spinal motor neurons was reduced by mutant SOD1 action and castration in mice. AR expression was previously described in motor neurons [39]; however, we show that AR is predominantly expressed by spinal motor neurons, but not astrocytes and microglia. Downregulation of AR in the gonads occurs in response to circulating androgen depletion by castration [40], consistent with our findings in the spinal cord. Our discovery of progressive AR depletion in spinal cords in SOD1^G93A^ mice from disease onset is a novel finding. While this could reflect motor neuron loss, reduced expression of AR on spinal motor neurons suggests a potential pathological role in neurodegeneration in these mice. Pathogenic expansions in AR leading to its inactivation causes lower motor neuron loss and muscle wasting in spinal bulbar muscular atrophy (SBMA) or Kennedy’s disease [41]. Pathogenic-expanded AR is linked to neurodegeneration by loss of normal AR trophic signalling, leading to transcriptional dysregulation of growth factors required for motor neuron survival such as brain-derived neurotrophic factor and transforming growth factor-β [42,43]. In addition, nuclear accumulation of toxic AR-polyglutamine expansions in motor neurons is implicated in SBMA [44]. It is noteworthy that we did not observe nuclear inclusions of AR in motor neurons of mutant SOD1 mice, implying a loss-of-function of AR in ALS. We therefore propose that AR reduction in spinal motor neurons may contribute to selective neuronal susceptibility in ALS. If supported, then ALS and SBMA could share a common disease pathway mediated by disruption of AR expression and function in spinal motor neurons.

## Conclusions

In conclusion, we demonstrate that stimulation of thymic function and CD4+ T cell production in mutant SOD1 mice does not delay disease onset and progression, in contrast to studies using passive transfer of Tregs. This may result from an inability of thymic activation induced by castration to increase neuroprotective Treg number due to limited IL-2 availability. Despite this, M1 microglia activation was reduced by this approach, although not conferring clinical benefit. Interestingly, we have discovered that AR is depleted in spinal motor neurons during the disease course of mutant SOD1 mice, suggesting a potential convergence of pathogenic mechanisms in ALS and SBMA that warrants further investigation.

## Abbreviations

ALS: amyotrophic lateral sclerosis
AR: androgen receptor
Arg1: arginase 1
CNS: central nervous system
Cx: castrated
FCS: foetal calf serum
iNOS: inducible nitric oxide synthase
GFAP: glial fibrillary acidic protein
PFA: paraformaldehyde
SBMA: spinal bulbar muscular atrophy
SOD1: superoxide dismutase 1
Tregs: T regulatory cells
WT: wild type
